# Investigating what felt shapes look like

**DOI:** 10.1177/2041669515627948

**Published:** 2016-01-27

**Authors:** Sam Clarke

**Affiliations:** St Anne’s College, University of Oxford, UK

**Keywords:** Molyneux’s question, inference, spatial representation, cross-modal perception, vision, touch

## Abstract

A recent empirical study claims to show that the answer to Molyneux’s question is negative, but, as John Schwenkler points out, its findings are inconclusive: Subjects tested in this study probably lacked the visual acuity required for a fair assessment of the question. Schwenkler is undeterred. He argues that the study could be improved by lowering the visual demands placed on subjects, a suggestion later endorsed and developed by Kevin Connolly. I suggest that Connolly and Schwenkler both underestimate the difficulties involved in rectifying the study they seek to fix. The problem is that the experimental paradigm under consideration fails to account for the role that rational inference plays in newly sighted subjects’ ability or inability to recognize spatial properties across modalities. Since answering Molyneux’s question requires establishing whether spatial properties can be recognized, across modalities, by newly sighted subjects without recourse to rational inference, this is a problem. Indeed, it is a problem that may be worsened by Schwenkler and Connolly’s suggestions regarding the lowering of visual demands on subjects in cross-modal matching tasks.

## Molyneux’s Question as an Experiment

Molyneux’s question (Locke, 1678/2008) can be understood in various different ways. On a standard reading, however, the question concerns whether vision and touch share a common spatial structure. It asks whether a congenitally blind subject, able to recognize felt shapes, would be able to recognize shapes solely on the basis of their clearly seen appearance if her sight were suddenly cured. The thought is this: If she could immediately recognize shapes visually, then this would have to be because of commonalities in the spatial structure of her novel visual experiences and her more familiar haptic experiences. Why? Because the subject lacks further resources upon which to make her identifications: She has had no chance to learn how felt shapes look, and she is (by stipulation) blocked from inferring^1^ an answer. Consequently, her identification of seen shapes would have to be something like her ability to recognize that a seen event is simultaneous with, or of equal duration to, a felt event; something that is, intuitively, an unlearnt capacity entailed by the common temporal structure of her perceptual experiences in either modality.

A recent empirical study (Held et al., 2011) purported to establish a negative answer to Molyneux’s question, however. It purported to establish that whatever commonalities exist between our visual and haptic experience of space, these provide an insufficient basis for making cross-modal identifications and that our cross-modal grasp of space is, therefore, quite unlike our cross-modal grasp of simultaneity or duration discussed earlier. To establish this, Held et al. examined five patients soon after surgery that had been performed to cure their congenital blindness. Patients were examined on:

1. TT tasks—where patients were asked to match a felt shape with an identical one of two subsequently felt shapes.
2. VV tasks—where patients were asked to match a seen shape with an identical one of two subsequently seen shapes.
3. TV tasks—where patients were asked to match a felt shape with an identical one of two subsequently seen shapes.

While patients’ performed well in TT and VV tasks—suggesting they were able to see and feel the objects—they performed poorly in TV tasks. Apparently, an ability to recognize felt shapes visually was something patients had not yet learnt to do.

If correct, Held et al.’s conclusion could have far-reaching philosophical (Eilan, 1993), developmental (cf. Spelke & Van de Walle, 1993) and architectural implications (cf. Matthen, 2014). It may be premature, however. A capacity to make gross same–different judgements does not imply a perception of figure (Evans, 1985). Successfully making same–different judgements need only involve one’s recognizing rough similarities or differences in low-level features perceived—for example, seen colors and shadows. It does not require that these perceptible features be perceived as the distinct facets of unified objects suitable for cross-modal comparison. Taking this as his starting point, Schwenkler (2012, 2013) has provided compelling reasons to think that patients in Held’s study probably succeeded in VV tasks despite “seeing” the world as a disunified array of features (edges, colors, etc.) and were thereby prevented from succeeding in TV tasks by their continued inability to visually perceive the bounded objects of cross-modal comparison, as such. This leaves the study’s findings largely orthogonal to the question of whether sight and touch share a common spatial structure: Molyneux remains unsatisfied.

Could Held’s experiment be rectified in this regard? A number of theorists, writing in this journal, have thought so: Schwenkler (2012, 2013) and Connolly (2013) have proposed and defended ingenious ways of lowering the visual demands placed on subjects during testing, such that they may be fairly tested soon, or even immediately, after corrective surgery has been completed to enable vision in the congenitally blind. In the remainder of this article, I want to raise a concern with these approaches that has been neglected in recent discussions.

## A Challenge

It is noteworthy that Held’s study could not have conclusively demonstrated a common spatial structure to vision and touch, even if newly sighted subjects had succeeded in TV tasks. Reasons for why this should be date back to the observations of Leibniz (1765/1981). He noted that Molyneux’s subject might successfully infer cross-modal identities. For example, in the case of the subject’s telling globe from cube, Leibniz noted the possibility that the subject might visually identify the objects by reflecting on their differing axes of symmetry—comparing the cube’s four (when seen face on) with the globe’s infinitely many (p. 135)—rather than their spatial structure in experience.

**Figure 1.**
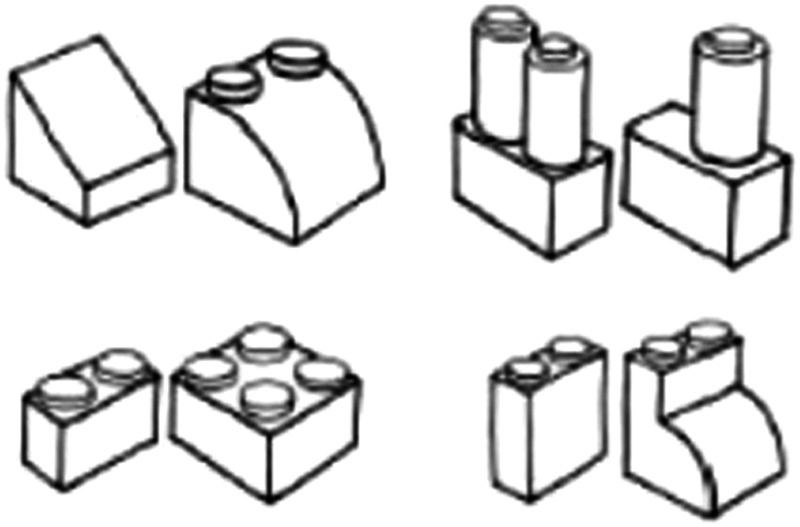
Example stimuli used by Held et al. (2011). Reprinted by permission from Macmillan Publishers Ltd: *Nature Neuroscience* 14(5), 2011.

Similar considerations apply to the Lego shapes used by Held et al. (Figure 1). It is conceivable that subjects might have recognized that one felt shape had many edges and thereby identified it with a busy area of their visual field, or tactually recognized that a given Lego brick is capped by roughly *x* number of identical studs and then identified it with roughly *x* number of identical looking (if disunified and ambiguous) features in some area of their visual field. Indeed, even if subjects were to be tested on pairs of shapes that mirrored one another’s form exactly, it is still possible^2^ that, given sufficient reasoning skills, they might correctly predict where larger quantities of seen features ought to be relative to smaller quantities of seen features, relative to their body. For instance, they might note that one shape had more edges and corners to the left side of their body, while the other had more to the right. None of this would require an appreciation of seen objects’ bounded form and none of it would require a common structure to their visual and haptic experience of space.

This is concerning. Fine et al. (2003) examined a once blind, but now “cured,” subject MM’s ability to visually interpret objects’ form, finding that, even 2 years after corrective surgery, MM’s limited ability to do this still “seemed to be based on explicit reasoning” (p. 915). Indeed, subject MM himself, described his struggle with the visual perception of objects’ bounded form as follows: “The difference between today and over two years ago is that I can better guess at what I am seeing. What is the same is that I am still guessing” (p. 916).

The worry this raises is that a newly sighted subject who succeeds in a Molyneux’s-style cross-modal matching task might only do so on the basis of a capacity for rational inference, rather than the recognition of a familiar spatial structure in experience. As such, Held et al.’s experimental paradigm seems doubly inadequate, incapable of delivering either a positive or negative answer to Molyneux’s question with any certainty.

This is troubling for Schwenkler and Connolly. Both propose that lowering visual demands on newly sighted subjects might enable fairer testing soon, or even immediately, after corrective surgery, *despite* subjects’ continued visual deficits. But, it is hard to see how this helps matters, in and of itself. Continued failures in TV tasks may reflect the continued visual deficits and abnormalities of newly sighted subjects’ vision, rather than the unfamiliar structure of normal visual perception. This might be so no matter how low visual demands are made. Meanwhile, successes may reflect the use of subjects’ intellect when inferring cross-modal identities in TV tasks, rather than commonalities in the spatiality of their experience; a cogent worry because, in lowering visual demands, inferential demands would seem to be lowered correspondingly.

For example, Schwenkler and Connolly propose that subjects might be tested with simple two-dimensional shapes instead of the three-dimensional Lego bricks pictured earlier. Since the newly sighted struggle with depth perception, it seems reasonable to suppose that the subjects might perform better in cross-modal matching tasks when asked to consider only simple squares and circles (cf. Connolly, 2013). But, it would then be unclear whether increased performance merely reflected the lower intellectual demands now placed on newly sighted subjects’ inferring cross-modal identities. After all, the subjects in such studies would now only need to recognize that four disunified features “fit” the felt square better than the felt circle^3^; a task placing significantly lower demands on subjects’ cognitive resources than a similar comparison of the Lego bricks used by Held et al.

Similar considerations also apply to a second suggestion made by Schwenkler for lowering visual demands. He proposes that subjects might have performed better in TV tasks if objects were presented to them in motion, citing a number of studies in support of the idea that this would help subjects to bind disunified features presented in visual experience into coherent wholes (2013). But this much is questionable: The relative motion of objects would plausibly only help them to distinguish the facets under consideration; for example, that the four visible sides (whether unified or disunified in experience) relate to the object of cross-modal comparison, against a backdrop of unmoving features, thereby aiding its inferred identification.

These considerations pose a problem for the empirical adjudication of Molyneux’s question using newly sighted subjects with surgically restored vision. Delivering a negative answer to the question would require establishing that subjects were not prevented from visually identifying the shapes by continued perceptual deficits, but how this might be determined remains unclear. After all, successfully identifying the bounds of an object may still rely on explicit reasoning, as suggested by Fine et al.’s study discussed earlier, rather than the binding of distinct features in experience. Conversely, establishing a positive answer to Molyneux’s question would require demonstrating that newly sighted subjects could visually identify felt shapes noninferentially. This would be problematic because lowering the visual demands placed on newly sighted subjects (as we must) would lower inferential demands correspondingly. There might be various ways of ruling inferential explanations out in either case—after all, perceptual recognition is characteristically fast, compared with rational inference, judgement independent in a way that rational inference is not, and so forth (Fodor, 1983). Researchers would do well to consider these characteristic differences in future adaptations of Held et al.’s experimental paradigm.

## Notes

1 By “inference” I mean a kind of reasoning, the conclusions of which are rationally evaluable. This distinguishes “inference” from, say, the operations of a subject’s visual system (cf. Fodor, 1983).

2 In principle—in reality, even normally perceiving subjects seem to struggle to make cross-modal identifications among mirrored shapes (Norman et al., 2004).

3 See Berkeley (1709/2014, §142) for a related point.
